# Noisy Light Augments the Na^+^ Current in Somatosensory Pyramidal Neurons of Optogenetic Transgenic Mice

**DOI:** 10.3389/fnins.2020.00490

**Published:** 2020-05-20

**Authors:** Pedro Mabil, Nayeli Huidobro, Oswaldo Torres-Ramirez, Jorge Flores-Hernandez, Amira Flores, Ranier Gutierrez, Elias Manjarrez

**Affiliations:** ^1^Laboratory of Integrative Neurophysiology, Instituto de Fisiología, Benemérita Universidad Autónoma de Puebla, Puebla, Mexico; ^2^Decanato de Ciencias Biológicas, Universidad Popular Autónoma del Estado de Puebla (UPAEP), Puebla, Mexico; ^3^Laboratory of Neuromodulation, Instituto de Fisiología, Benemérita Universidad Autónoma de Puebla, Puebla, Mexico; ^4^Departamento de Farmacología, CINVESTAV-IPN, Mexico City, Mexico

**Keywords:** stochastic resonance, optogenetics, noise, opto nongenetics, transcranial random noise stimulation, optonongenetic, photostimulation, tRNS

## Abstract

In previous reports, we developed a method to apply Brownian optogenetic noise-photostimulation (BONP, 470 nm) up to 0.67 mW on the barrel cortex of *in vivo* ChR2 transgenic mice. In such studies, we found that the BONP produces an increase in the evoked field potentials and the neuronal responses of pyramidal neurons induced by somatosensory mechanical stimulation. Here we extended such findings by examining whether the same type of BONP augments the Na^+^ current amplitude elicited by voltage-clamp ramps of dissociated pyramidal neurons from the somatosensory cortex of ChR2 transgenic and wild type mice. We found that in all neurons from the ChR2 transgenic mice, but none of the wild type mice, the peak amplitude of a TTX-sensitive Na^+^ current and its inverse of latency exhibited inverted U-like graphs as a function of the BONP level. It means that an intermediate level of BONP increases both the peak amplitude of the Na^+^ current and its inverse of latency. Our research suggests that the impact of BONP on the Na^+^ channels of pyramidal neurons could be associated with the observed augmentation-effects in our previous *in vivo* preparation. Moreover, it provides caution information for the use of an appropriate range of light intensity, <0.67 mW, which could avoid opto non-genetics (also termed “optonongenetic”) related responses due to light-induced temperature changes.

## Introduction

Medina et al. (2012) demonstrated for the first time that the application of electrical noise on the primary somatosensory cortex improves the artificial tactile perception in monkeys. A subsequent study in humans showed that the sensory perception could also be enhanced via the stochastic resonance mechanism (Moss et al., 2004; Faisal et al., 2008; McDonnell and Ward, 2011; McDonnell et al., 2015) when an intermediate level of transcranial electrical noise stimulation is applied (Van der Groen and Wenderoth, 2016). Furthermore, previous studies in artificial lipid bilayers reported that the application of an intermediate level of electrical noise augments the current magnitude throughout alamethicin channels (Bezrukov and Vodyanoy, 1995, 1997b). As a first step, these findings motivated our laboratory to explore some indirect mechanistic questions regarding the effects of electrical noise on pyramidal neurons (Remedios et al., 2019). In such a previous study, we examined whether the direct-application of electrical noise on dissociated pyramidal neurons from the somatosensory cortex can also improve the Na^+^ current amplitude elicited by voltage-clamp ramps (Remedios et al., 2019). We found that the application of an intermediate level of Brownian electrical noise stimulation on dissociated pyramidal neurons can produce an augmentation in the Na^+^ current amplitude and a modulatory effect on its latency (Remedios et al., 2019). Likewise, we demonstrated, experimentally and theoretically, with a Hodgkin-Huxley model, that such augmentation-effects are related to the impact of electrical noise on the kinetics of activation and inactivation of the Na^+^ channels (Remedios et al., 2019). Therefore, a natural question derived from these previous findings is whether the random nature of electrical noise is essential for the augmentation-phenomenon in the Na^+^ current or whether the electrical nature of the stimuli is responsible for such augmentation in the Na^+^ current. We hypothesized that if the random nature of the noise is the essential element in the observed augmentation-effect, then other physical sources of noise could also produce similar augmentation-effects on the Na^+^ current. Therefore, the present study aimed to examine whether the application of BONP on dissociated pyramidal neurons obtained from ChR2 transgenic mice could also produce an augmentation-effect in the Na^+^ current amplitude and a modulatory effect on its latency. Our results are consistent with the idea that an inherent action of random noise is related to the observed augmentation-effects, thus in line with the previous hypothesis claiming that the stochastic resonance is an inherent property of rate-modulated random series of events (Bezrukov, 1998). Our results provide support to previous observations, claiming that the short-duration BONP on the barrel cortex of ChR2 transgenic mice can increase the evoked field potentials and the firing frequency response of pyramidal neurons to somatosensory mechanical stimulation (Huidobro et al., 2017, 2018). Finally, to exclude an optonongenetic effect due to temperature changes induced by the delivered light (Ait Ouares et al., 2019; Ghirga et al., 2020) we examined whether our intensities of BONP up to 0.67 mW produce similar effects in wild type mice. Our results provide information for an appropriate range of light intensity to avoid the optonongenetic responses due to light-induced temperature changes.

## Materials and Methods

Experiments were performed in eleven transgenic mice (weighing 35 ± 3 g, mean ± SD) and in two wild type mice (weighting 38 and 43 g). The transgenic mice were Thy1-ChR2-YFP, expressing the light-activated ion channel Channelrhodopsin-2 (ChR2, obtained from the green alga Chlamydomonas reinhardtii), and fused to the Yellow Fluorescent Protein (ChR2-YFP) under the control of the mouse thymus cell antigen 1 (Thy1) promoter. The Thy1-ChR2-YFP animals were acquired from Jackson Labs (JAX United States) and raised in the animal facility of the CINVESTAV-IPN, Mexico. We performed the polymerase chain reaction (PCR)-based genotyping in all the mice. The animals had free access to food and water and were kept in rooms with controlled temperature and light exposure (lights on at 6 a.m. and lights off at 6 p.m.). We followed the guidelines contained in the “Norma-Oficial-Mexicana-NOM-062-ZOO-1999,” the European communities council directive of 24-November-1986 (86/609/EEC), and the Guide from the National Institutes of Health for the Care and Use of Laboratory Animals (85–23, revised in 1985). All our protocols were approved by the local ethics committee (CICUAL-Proyectos: 00489, 1001189699-UALVIEP-20/2) from the Benemérita Universidad Autónoma de Puebla.

**FIGURE 1:**
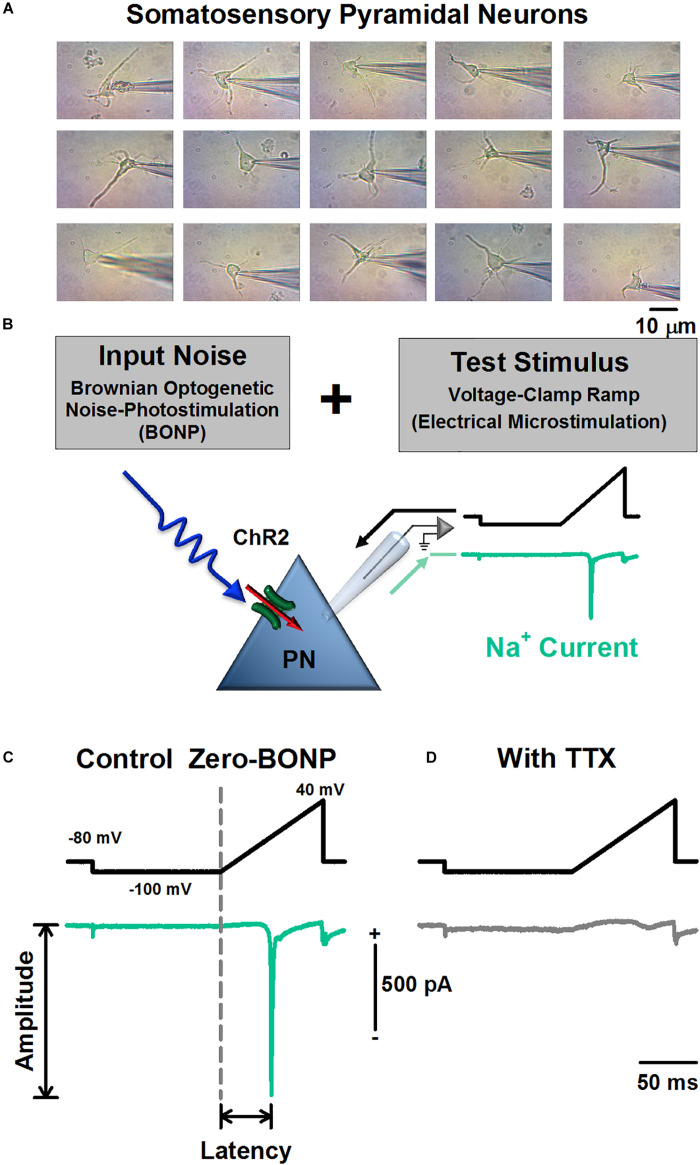
Scheme of the experimental arrangement for the Brownian optogenetic noise photostimulation (BONP) and the electrical microstimulation with voltage-clamp ramps. **(A)** Pictures of a sample of 15 pyramidal neurons (PN) from the somatosensory cortex recorded from five Thy1-ChR2-YFP transgenic mice. **(B)** The experimental arrangement of the BONP (Input noise, from 0 to 0.67 mW, 470 nm) applied during the electrical microstimulation with voltage-clamp ramps (test stim). **(C)** The peak amplitude and latency of the Na^+^ current. **(D)** Recording showing how the application of TTX abolished the Na^+^ current. The use of TTX was made at the end of each cellular record to verify that the Na^+^ current was TTX sensitive.

Experiments were performed in 25 dissociated pyramidal neurons from the somatosensory cortex of 11 Thy1-ChR2-YFP transgenic mice. Other control experiments were performed in 10 pyramidal neurons from the somatosensory cortex of two wild type mice. We used halothane to anesthetize the mice before the decapitation. To acutely dissociate the pyramidal neurons, we followed the same procedures described in previous articles (Bargas et al., 1994). Briefly, the brain tissue was placed in a cold solution of isethionate with low calcium. Coronal slices of 350 μM from the barrel somatosensory cortex were obtained. After at least 1 h of the slices incubation, every slice was placed in enzymatic digestion. After the enzymatic digestion, the tissue was washed and mechanically dissociated with Pasteur pipettes. We obtained a cell suspension with the acutely isolated neurons. We used an inverted microscope to identify live isolated pyramidal-cells. Figure 1A illustrates a sample of 15 pyramidal somatosensory cells. The dissociation methods and solutions were identical to those employed in a previous study (Remedios et al., 2019).

**FIGURE 2:**
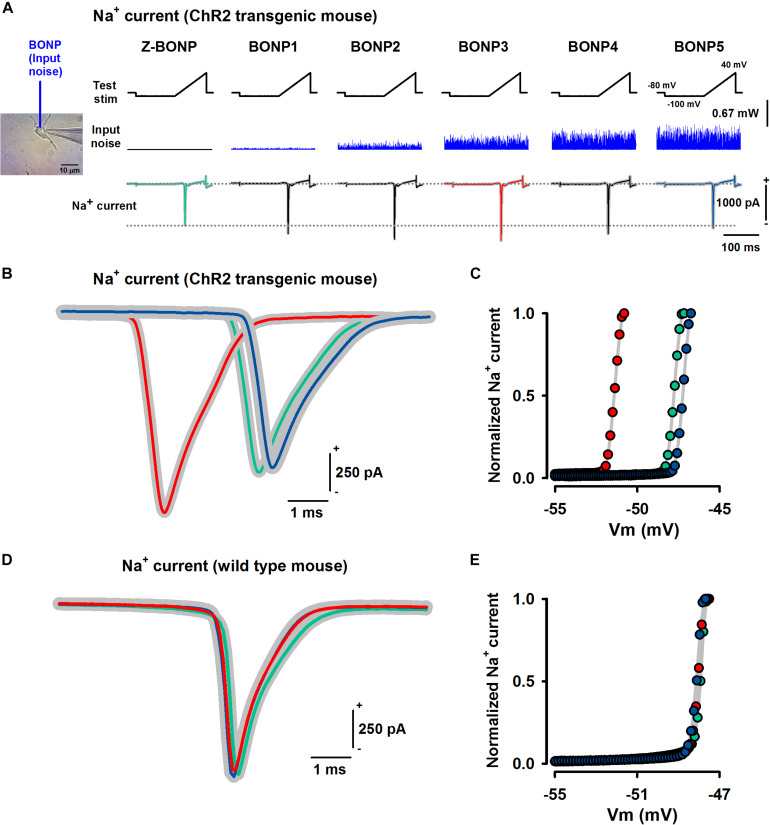
Effects of BONP on the peak amplitude of Na^+^ currents elicited by voltage-clamp ramps in dissociated pyramidal neurons from the somatosensory cortex of Thy1-ChR2-YFP and wild type mice. **(A)** Voltage-clamp ramps (test stimulation) and the associated Na^+^ currents during the application of different levels of BONP (input noise) on the pyramidal cell of a Thy1-ChR2-YFP mouse. Note the change in the Na^+^ current amplitude and its latency for an intermediate intensity of BONP (occurring at BONP3, in this case). **(B)** Superimposed traces of three Na^+^ currents for three levels of noise, zero BONP (green), optimal BONP (red), and high BONP (blue) for a Thy1-ChR2-YFP mouse (“ChR2 transgenic mouse”). Similar results were obtained in 25 pyramidal neurons of 11 transgenic mice. **(C)** The voltage activation curves for the Na^+^ currents illustrated in **(B)**. Note the shift in the curve associated with the optimal BONP (red symbols), where Vm is the membrane potential. **(D,E)** The same as **(B,C)**, but for a wild type mouse. Similar results were obtained in 10 pyramidal neurons of two wild type mice. Note that the Na^+^ current and its voltage activation curves were not affected by the BONP. The color traces in green, red, and blue represent the mean value of the recorded Na^+^ currents. Whereas, the gray shadow around these traces is the standard deviation for the recorded currents in each case.

The pyramidal neurons were recorded in whole-cell mode and were microstimulated with six groups of 10 voltage-clamp ramps of 100 ms duration, from –100 mV to + 40 mV, with a holding potential of –80 mV and a pre-pulse duration of 125 ms from −80 mV to –100 mV. At the end of each ramp, the voltage returned to the holding potential (Figure 2).

The dissociated pyramidal neurons from both the transgenic and the wild type mice were stimulated with BONP, which consisted of blue light of 470 nm. We applied the BONP in the optical power range from 0 to 0.67 mW using an optic fiber of 200 micrometers positioned close to the cell. The optic fiber had a numerical aperture of 0.39 (starter kit from Thorlabs) and was positioned with a micromanipulator at an angle of 45°. The experimental arrangement is illustrated in Figure 1B. The BONP stimulus exhibited a flashing-like behavior with random fluctuations in the intensities of light. The power spectrum of this light was Brownian in the frequency range from 0 to 5,000 Hz (see power spectrum of BONP in previous studies, Huidobro et al., 2017, 2018). The optical power of the blue light was measured with an optical power meter PM100D and sensor S150C from Thorlabs. Our stimulation protocol consisted of a series of six voltage-clamp ramps for every level of BONP (Figure 2A). We applied the zero-noise of BONP (control, Z-BONP). We also used five other different levels of BONP (BONP1, BONP2, BONP3, BONP4, BONP5). Every level of BONP was continuously applied during all the patch-clamp protocol of five voltage-clamp ramp microstimulations.

**FIGURE 3:**
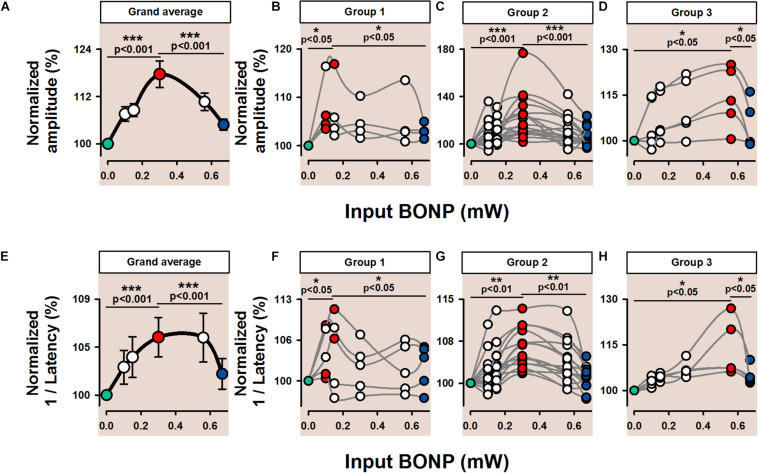
Pooled data are showing the effects of BONP on the peak amplitude and inverse of latency of Na^+^ currents elicited by voltage-clamp ramps in the Thy1-ChR2-YFP mice. **(A)** The grand average of the normalized peak amplitude of the Na^+^ current (%, percent of change) vs. the input BONP for 25 pyramidal neurons from the somatosensory cortex of eleven Thy1-ChR2-YFP transgenic mice. **(B)** Group 1, pyramidal neurons are exhibiting a similar profile in which the optimal BONP was around 0.1 mW. Error bars indicate standard deviation (SD). **(C)** Group 2, pyramidal neurons are exhibiting a similar profile in which the optimal BONP was around 0.3 mW. **(D)** Group 3, pyramidal neurons are showing a similar pattern in which the optimal BONP was ∼0.58 mW. **(E–H)** The same as **(A–D)** but for the inverse of latency of the Na^+^ current. All these graphs illustrate that intermediate levels of BONP, in the range from 0.1 to 0.67 mW, produced a statistically significant increase in the peak amplitude of the Na^+^ current and its inverse of latency in all transgenic mice (*p* < 0.05). The colors indicate three conditions: during zero BONP (green), optimal BONP (red), and high BONP (blue). *p < 0.05, **p < 0.01, and ***p < 0.001.

Furthermore, to avoid adaptation, rest intervals of 3 s were included between the BONP levels. Because the noise levels were determined by the output of an analogic WaveTek noise generator, the choice of the BONP levels was not uniform. However, to avoid that the optimal BONP could not be covered, we presented such BONP levels in a pseudo-randomized fashion. Moreover, the values of the Na^+^ current peak amplitude, illustrated in Figure 3, were joined with smooth curved lines by using the SigmaPlot software, without any interpolation.

To test for any statistical difference in the peak amplitude of the Na^+^ current and its inverse of latency, we compared them in the following conditions: Z-BONP vs. BONP1, Z-BONP vs. BONP2, Z-BONP vs. BONP3, Z-BONP vs. BONP4, Z-BONP vs. BONP5. We also compared BONP1, BONP3, or BONP4 vs. BONP5. We used the non-parametric pairwise Signed-Rank Tests to examine the statistical significance.

We compared the abovementioned conditions of BONP in all the animals under the null hypothesis that the differences of the means between such conditions were zero. Due to the multiple comparisons, we used a corrected Bonferroni adjustment. Error bars indicate standard deviation. We considered the comparisons significant if *p* < 0.05. Data obtained from each neuron are available from the authors upon request.

## Results

We applied different levels of BONP to 25 pyramidal cells from the somatosensory cortex of eleven Thy1-ChR2-YFP transgenic mice. Moreover, we used the same levels of BONP to be applied to the other ten pyramidal cells from the somatosensory cortex of two wild type mice. We verified that all the neurons were pyramidal, as illustrated in Figure 1A. In particular, as shown in Figure 1C, we analyzed the effects of BONP on the peak amplitude and the inverse of latency of Na^+^ currents. We found statistically significant changes produced by BONP in all transgenic mice (*p* < 0.05) but not in the wild type mice, as will be described in the following paragraphs. We verified that Na^+^ currents were abolished by TTX (Figure 1D).

Figure 2A shows the effects of six levels of noise-photostimulation (Z-BONP, BONP1, BONP2, BONP3, BONP4, BONP5) on the peak amplitude of the Na^+^ current elicited by the voltage-clamp ramp microstimulation. We presented such BONP levels in a pseudo-randomized fashion, but for clarity, in this Figure 2A, they are illustrated in sequence.

Application of an optimal BONP level reduced the Na^+^ current latency, along with an increased Na^+^ current peak (Figure 2B). The color traces in green, red, and blue represent the mean value of the recorded Na^+^ currents. Whereas, the gray shadow around these traces is the standard deviation for the recorded currents in each case. The green traces represent the control, and the blue traces the high level of noise. The red traces in Figures 2A,B were obtained in the condition of optimal BONP. Note that there is an intermediate and an optimal level of BONP, in for which the peak amplitude of the Na^+^ current is enhanced. This result was reproduced in all the 25 pyramidal somatosensory neurons tested. We found that in all transgenic mice, the gain for the Na^+^ current under the optimal BONP relative to zero noise was 317 ± 268 pA for its peak amplitude and 2.54 ± 1.9 ms for its latency shift. The differences were statistically significant, *p* < 0.001, see percentages of change in Table 1. Figure 2C also shows that the voltage activation curves of the Na^+^ current exhibit a shift for intermediate intensities of BONP. This behavior was also reproducible for all the pyramidal neurons obtained from the transgenic animals.

**TABLE 1:** Statistical analysis for the comparison of the effects of BONP in the normalized amplitude and the normalized 1/latency of the Na^+^ current, where Mdn is the median.

**Signed-rank test (Normalized Amplitude)**																		
**ZN-BONP vs. BONP1**							**ZN-BONP vs. BONP2**						**ZN-BONP vs. BONP3**					
	**ZN-BONP Mdn**	**BONP1 Mdn**	**T**	***z***	***r***	***p***	**ZN-BONP Mdn**	**BONP2 Mdn**	**T**	***z***	***r***	***p***	**ZN-BONP Mdn**	**BONP3 Mdn**	**T**	***z***	***r***	***p***
Grand average	100.00	105.85	30.00	–3.57	–0.71	0.0003	100.00	105.73	8.00	–4.16	–0.83	0.00003	100.00	110.65	1.00	–4.35	–0.87	0.00001
Group 1	100.00	105.34	0.00	–1.83	–0.61	0.034	100.00	104.71	0.00	–1.83	–0.61	0.034	100.00	103.78	0.00	–1.83	–0.61	0.034
Group 2	100.00	106.12	14.00	–2.79	–0.68	0.005	100.00	106.01	5.00	–3.23	–0.81	0.001	100.00	119.04	0.00	–3.52	–0.88	0.0004
Group 3	100.00	101.66	4.00	–0.94	–0.42	0.17	100.00	103.45	1.00	–1.75	–0.78	0.04	100.00	106.52	1.00	–1.75	–0.78	0.04
	**ZN-BONP vs. BONP4**						**ZN-BONP vs. BONP5**						**BONP1 vs. BONP5, BONP3 vs. BONP5, or BONP4 vs. BONP5,**					
	**ZN-BONP Mdn**	**BONP4 Mdn**	**T**	***z***	***r***	***p***	**ZN-BONP Mdn**	**BONP5 Mdn**	**T**	***z***	***r***	***p***	**BONP1, BONP3, or BONP4, Mdn**	**BONP5 Mdn**	**T**	***z***	***r***	***p***

Grand average	100.00	106.73	13.00	–4.02	–0.8	0.0005	100.00	102.91	50.00	–3.03	–0.61	0.002	110.65	102.91	2.00	–4.32	0.86	0.00001
Group 1	100.00	102.95	0.00	–1.83	–0.61	0.034	100.00	102.90	0.00	–1.83	–0.61	0.034	105.34	102.90	0.00	–1.83	–0.61	0.034
Group 2	100.00	106.63	7.00	–3.52	–0.88	0.002	100.00	102.91	22.00	–2.38	–0.59	0.017	119.04	102.91	0.00	–3.52	–0.88	0.0004
Group 3	100.00	113.11	0.00	–2.00	–0.89	0.02	100.00	99.57	6.00	–0.4	–0.18	0.34	113.11	99.57	0.00	–2.02	–0.9	0.02

**Signed-rank test (Normalized 1/Latency)**																		
**ZN-BONP vs. BONP1**							**ZN-BONP vs. BONP2**						**ZN-BONP vs. BONP3**					
	**ZN-BONP Mdn**	**BONP4 Mdn**	**T**	***z***	***r***	***p***	**ZN-BONP Mdn**	**BONP5 Mdn**	**T**	***z***	***r***	***p***	**BONP1, BONP3, or BONP4, Mdn**	**BONP5 Mdn**	**T**	***z***	***r***	***p***

Grand average	100.00	102.18	29.00	–3.59	–0.72	0.0003	100.00	102.97	23.00	–3.75	–0.75	0.0001	100.00	104.86	5.00	–4.23	–0.85	0.00002
Group 1	100.00	103.78	0.00	–2.02	–0.91	0.022	100.00	106.72	3.00	–2.21	–0.99	0.02	100.00	102.52	3.00	–1.21	–0.54	0.11
Group 2	100.00	100.85	23.00	–2.10	–0.68	0.01	100.00	101.75	9.00	–2.89	–0.75	0.002	100.00	104.86	0.00	–3.41	–0.88	0.0006
Group 3	100.00	103.14	0.00	–2.02	–0.91	0.022	100.00	104.64	0.00	–2.02	–0.91	0.022	100.00	106.39	0.00	–2.02	–0.91	0.022

	**ZN-BONP vs. BONP4**						**ZN-BONP vs. BONP5**						**BONP2 vs. BONP5, BONP3 vs. BONP5, or BONP4 vs. BONP5,**					
	**ZN-BONP Mdn**	**BONP4 Mdn**	**T**	***z***	***r***	***p***	**ZN-BONP Mdn**	**BONP5 Mdn**	**T**	***z***	***r***	***p***	**BONP1, BONP3, or BONP4, Mdn**	**BONP5 Mdn**	**T**	***z***	***r***	***p***

Grand average	100.00	104.44	19.00	–3.86	–0.77	0.0001	100.00	101.98	46.00	–2.97	–0.59	0.003	104.86	101.98	33.00	–3.48	0.7	0.0004
Group 1	100.00	101.24	4.00	–0.94	–0.42	0.17	100.00	103.73	1.00	–1.46	–0.65	0.07	106.72	103.73	1.00	–2.02	–0.91	0.04
Group 2	100.00	102.94	5.00	–3.12	–0.81	0.001	100.00	101.24	30.00	–1.71	–0.44	0.044	104.86	101.24	0.00	–3.41	–0.88	0.0006
Group 3	100.00	107.35	0.00	–2.02	–0.91	0.022	100.00	103.81	0.00	–2.02	–0.91	0.022	107.35	103.81	0.00	–2.02	–0.91	0.022

**Because the data were not normally distributed (Kolmogorov-Smirnov normality test, P < 0.05) and had not homogeneity of variances (Levene test, P < 0.05), we performed several pairwise Signed-Rank Tests, under the null hypothesis that the differences between the aforementioned conditions were zero. The test of significance was one-tailed. Values of P < 0.05 were considered significant.**

In contrast, for the wild type mice, we did not observe significant changes in the peak amplitude (Figure 2D), latency (Figure 2D), or voltage activation curves (Figure 2E) of the Na^+^ current. This behavior was reproducible for all the ten pyramidal neurons obtained from the wild type mice. Data analysis and graphs derived from every single neuron are available from the authors upon request.

Figure 3 shows pooled data of our results obtained from the ChR2 transgenic animals (see also Table 1 for a detailed statistical analysis). Figure 3A is the grand average for the percentage of change in the peak amplitude of the Na^+^ current vs. the different levels of BONP. Note that the Na^+^ current amplitude follows an inverted U-like shape as a function of the BONP level for all the 25 pyramidal neurons. Note the highly statistically significant difference between the Na^+^ current amplitude at Z-BONP (green circle), or high noise (blue circle), vs. the Na^+^ current amplitude during an intermediate intensity of BONP (red circle). The non-parametric pairwise Signed-Rank Tests uncovered significant differences, as illustrated in Figure 3A and Table 1. In general, we found that an intermediate level of optimal BONP produced a statistically significant increase in the peak amplitude of the Na^+^ current in all transgenic mice (*p* < 0.001). To visualize the different ranges for the optimal BONP in our experiments we grouped the graphs according to their shape. In Figure 3B, we show a group of graphs for pyramidal neurons exhibiting a similar profile in which the optimal BONP was around 0.1 mW. In Figure 3C, we show another group of graphs for pyramidal neurons showing the same pattern in which the optimal BONP was ∼0.3 mW. Whereas in Figure 3D, we illustrate a group of graphs for pyramidal neurons exhibiting a similar profile in which the optimal BONP was around 0.58 mW.

We also examined whether the inverse of latency of the Na^+^ current also exhibits similar behavior as the peak amplitude of the Na^+^ current when different levels of BONP are applied. Figure 3 and Table 1 show such an analysis. Figure 3E is the grand average for the percentage of change of the inverse of latency of the Na^+^ current vs. the different levels of BONP. We also found that an intermediate level of BONP produces a statistically significant increase in the inverse of latency of the Na^+^ current in all transgenic mice (*p* < 0.001). To visualize the variability of optimal BONP measured, we plotted the inverse of latency of Na^+^ currents with a similar shape together. Figures 3F–H show the different groups obtained from the selection of graphs according to their response profile.

## Discussion

We found that the BONP produces an inverted U-like shape in the Na^+^ current amplitude and its inverse of latency as a function of the BONP level in somatosensory pyramidal neurons of Thy1-ChR2-YFP transgenic mice. It means that an intermediate level of BONP can increase the peak amplitude of the Na^+^ inward current and its inverse of latency of pyramidal neurons from the somatosensory cortex elicited by voltage-clamp microstimulation. The technique employed in the present article was based on two previous studies from our laboratory, in which we introduced a new type of brain stimulation with noisy light, termed BONP (Huidobro et al., 2017; Huidobro et al., 2018). Both studies provided support to the findings of the present article. The consistency between the inverted U-like behaviors of the neuronal responses in the *in vitro* and the *in vivo* preparations suggests that the observed behavior in the *in vivo* preparation could be due in part to the impact of BONP via the ChR2 channel on the Na^+^ current.

A future modeling study with Brownian optical noise using the interaction of light with ChR2 channels (see Grossman et al., 2011; Nikolic et al., 2013) will be necessary to examine mechanisms. Such a modeling study using BONP could help to answer three fundamental questions: (1) how does the BONP increase the excitability of Na^+^ channels? (2) why is there an optimal intermediate level of BONP? (3) how does the high level of BONP decreases the excitability of Na^+^ channels? A limitation of our study is that the responses to such questions are highly speculative. A possible answer to question 1 is that the BONP indirectly increases the excitability of Na^+^ channels via their action on the ChR2 channels, plausibly through their impact on the kinetics of activation and inactivation of the Na^+^ channels (Remedios et al., 2019). Our finding that the BONP produces a shift in the voltage activation curves in transgenic animals (Figure 2C) but not in wild type mice (Figure 2E) supports this possibility. In this context, the lower triggering potential associated with an optimal BONP could also be due to this shift in the voltage activation curve. Finally, a suggested response for questions 2 and 3 is that there is an intermediate level of BONP with an inverted U-like profile because the BONP is associated with a stochastic resonance-like phenomenon (Moss et al., 2004) occurring in the impact on BONP on the ChR2 and Na^+^ channels, as previously suggested in *in vivo* preparations (Huidobro et al., 2017, 2018).

Although we have not an explanation for the observation that the BONP stimulation shifts the activation time of Na^+^ currents in Thy1-ChR2-YFP transgenic mice, we can only speculate in this respect based on results from experiments in rat pyramidal neurons. Previously, we found a similar shift in the latency of the Na^+^ current, produced by the application of electrical noise in rat pyramidal neurons from the auditory and somatosensory cortex (Remedios et al., 2019). We found that the electrical noise stimulation affects the Na^+^ current latency and that such a shift is associated with the impact of noise on the kinetics of activation and inactivation of the Na^+^ channels. In such a study, we also reproduced the experimental results with a Hodgkin-Huxley neuron model, demonstrating at the mechanistic level that the impact of noise occurs on the kinetics of activation and inactivation of the Na^+^ channels. Because we found similar shifts in the Na^+^ currents produced by BONP, we could speculate that the BONP also changes the kinetics of activation and inactivation of the Na^+^ channels; however, it may occur indirectly throughout its impact on the ChR2 channels. Future modeling studies, based in the four-state ChR2-model by Grossman et al. (2011), for instance, will be necessary to examine such possibility.

From an integrative perspective, our results are consistent with previous studies showing that the electrical noise applied to the somatosensory cortex could be employed to improve tactile perception (Medina et al., 2012) and the augmentation of the brain function via brain-machine interfaces (Lebedev et al., 2018). Whereas at the cellular level, the present results are also consistent with other studies using alamethicin ion channels stimulated with electrical noise (Bezrukov and Vodyanoy, 1995, 1997a).

In the present research, we employed light instead of electrical or magnetic stimuli, because the light provides precise control of neuronal activity when it is applied in transgenic mice expressing ChR2 (Arenkiel et al., 2007). Specifically we used light given that it is becoming useful to employ it in animals with an injection of viral vectors that express light-sensitive proteins (Jackman et al., 2019). For instance, the use of gene regulatory sequences in viral vectors to target specific neuronal types could allow specific optogenetic-stimulation with the light of particular kinds of neurons in primates (El-Shamayleh and Horwitz, 2019), mice (Zelena et al., 2017), and rats (Pawela et al., 2016).

Although the most common types of light stimulation (also termed optogenetic stimulation) are the pulsed (light on-off), sinusoidal, and ramp light across a range of light intensities, we decided to use the BONP in our experiments. Such a decision was motivated by our successful results obtained in the *in vivo* preparation (Huidobro et al., 2017, 2018). However, we can not exclude the possibility that other optical wave-forms could also produce similar augmenting effects on the Na^+^ current amplitude. We did not perform such comparative experiments in the same cells given the difficulty to maintain for more prolonged periods the voltage-clamp recordings.

Because we aimed to examine the effects of optogenetic-noise stimulation on the Na^+^ current amplitude, we used electrical stimulation (voltage-clamp ramps) instead of pulses of optogenetic stimulation. On the other hand, it would be challenging to generate Na^+^ currents from “optical-ramps” or pulses of optogenetic stimulation. However, the noisy optogenetic-stimulation may improve the response of action potentials on single cells with a high spatial and temporal resolution. But this type of experiment could be implemented in future studies.

Recently, it was demonstrated that the light by itself is capable of modulating ion channels in wild type mice by transiently changing the temperature from 0.1 to 0.4°C, and this effect is different from one cell type to another in the brain (Ait Ouares et al., 2019). The discrepancy between the observations by Ait Ouares et al. (2019), and our results could be due to the different light intensities employed. In our experiments, in transgenic and wild type mice, we applied light with an optical power-range from 0 to 0.67 mW, whereas Ait Ouares et al. (2019) applied light at higher intensities of 1, 5, and 13 mW. Ait Ouares et al. (2019) demonstrated that tissue temperature linearly increases with light power reaching an average value of 0.03°C at 1mW and 0.4°C at 13 mW. Nevertheless, as illustrated in Figure 3 (see horizontal axis), we found that the optimal levels of BONP (red circles) for all our pyramidal cells were lower than 0.6 mW, i.e., around 0.1, 0.3, and 0.58 mW, as illustrated in the grouped graphs. Therefore, based on these comparisons, we could suggest that our results in transgenic mice are due to the effect of BONP on the Na^+^ channels but not to temperature changes. It means that the optogenetic stimulation can produce physiological responses in neurons not associated with light-induced temperature changes, at least at a range below 0.67 mW. In this context, our results contribute to the optogenetics field, stating that a way to avoid the “optonongenetic related responses” due to changes in temperature, the researchers should use light at least at a lower intensity than 0.67 mW. This is consistent with recent studies of calcium imaging and patch clamp experiments, showing that only the illumination higher than 1 mW causes an optonongenetic enhancement of network activity in cortical cultures of wild type mice (Ghirga et al., 2020). Future experiments in transgenic animals will be necessary to identify the exact intensity of light that produces both an optogenetic and a light-induced temperature change [e.g. see simulations by Shin et al. (2016)].

Our findings allow us to speculate that the intracortical-microstimulation could be combined with the BONP in awake transgenic-animals expressing ChR2. This could be beneficial for the development of new technologies for long-lasting intracortical microstimulation with brain-machine interfaces in animal models (Callier et al., 2015); which is of importance for future clinical applications in the human brain.

We conclude that there are intermediate intensities of BONP of 470 nm augmenting the peak amplitude of TTX-sensitive Na^+^ currents in pyramidal neurons from the somatosensory cortex of ChR2- transgenic mice. Such medium energies of BONP are in the range from 0.1 to 0.58 mW, and they do not produce effects on the Na^+^ current of pyramidal neurons in wild type mice. In this context, we demonstrate that the observed effects are not due to temperature changes produced by the stimulation with light. These conclusions provide support to previous experiments obtained from *in vivo* experiments and they provide caution information for the ranges of light intensity (lower than 0.67 mW) that could avoid the optonongenetic responses due to light-induced temperature changes.

## Data Availability Statement

The raw data supporting the conclusions of this article will be made available by the authors, without undue reservation, to any qualified researcher.

## Ethics Statement

The animal study was reviewed and approved by Consejo Institucional para el Cuidado y Uso de Animales de Laboratorio (CICUAL) from Benemérita Universidad Autónoma de Puebla.

## Author Contributions

EM conceived and designed the experiments, wrote the manuscript, and conceived the BONP method. PM, JF-H, AF, NH, OT-R, RG, and EM adapted the noise photostimulation to the *in vitro* setup, performed the whole-cell experiments and the authors revised and approved the manuscript. AF and NH performed the statistical analysis. PM and NH did all the analyses of the experiments. RG provided the Thy1-ChR2-YFP and wild type mice. EM supervised all aspects of the work.

## Conflict of Interest

The authors declare that the research was conducted in the absence of any commercial or financial relationships that could be construed as a potential conflict of interest.

## References

[B1] Ait Ouares K. Beurrier C. Canepari M. Laverne G. Kuczewski N. (2019). Opto nongenetics inhibition of neuronal firing. *Eur. J. Neurosci.* 49 6–26. 10.1111/ejn.14251 30387216

[B2] Arenkiel B. R. Peca J. Davison I. G. Feliciano C. Deisseroth K. Augustine G. J. (2007). In vivo light-induced activation of neural circuitry in transgenic mice expressing channelrhodopsin-2. *Neuron* 54 205–218. 10.1016/j.neuron.2007.03.005 17442243 PMC3634585

[B3] Bargas J. Howe A. Eberwine J. Cao Y. Surmeier D. J. (1994). Cellular and molecular characterization of Ca2+ currents in acutely isolated, adult rat neostriatal neurons. *J. Neurosci.* 14 6667–6686. 10.1523/jneurosci.14-11-06667.1994 7965068 PMC6577263

[B4] Bezrukov S. M. (1998). Stochastic resonance as an inherent property of rate-modulated random series of events. *Phys. Lett. A* 248 29–36. 10.1016/s0375-9601(98)00610-0

[B5] Bezrukov S. M. Vodyanoy I. (1995). Noise-induced enhancement of signal transduction across voltage-dependent ion channels. *Nature* 378 362–364. 7477370 10.1038/378362a0

[B6] Bezrukov S. M. Vodyanoy I. (1997a). Stochastic resonance in non-dynamical systems without response thresholds. *Nature* 385 319–321. 10.1038/385319a0 9002515

[B7] Bezrukov S. M. Vodyanoy I. (1997b). Signal transduction across alamethicin ion channels in the presence of noise. *Biophys. J.* 73 2456–2464. 10.1016/s0006-3495(97)78274-2 9370439 PMC1181147

[B8] Callier T. Schluter E. W. Tabot G. A. Miller L. E. Tenore F. V. Bensmaia S. J. (2015). Long-term stability of sensitivity to intracortical microstimulation of somatosensory cortex. *J. Neural Eng.* 12:056010. 10.1088/1741-2560/12/5/056010 26291448

[B9] El-Shamayleh Y. Horwitz G. D. (2019). Primate optogenetics: progress and prognosis. *Proc. Natl. Acad. Sci. U.S.A.* 116 26195–26203. 10.1073/pnas.1902284116 31871196 PMC6936537

[B10] Faisal A. A. Selen L. P. J. Wolpert D. M. (2008). Noise in the nervous system. *Nat. Rev. Neurosci.* 9 292–303. 18319728 10.1038/nrn2258 PMC2631351

[B11] Ghirga S. Pagani F. Rosito M. Di Angelantonio S. Ruocco G. Leonetti M. (2020). Optonongenetic enhancement of activity in primary cortical neurons. *J. Opt. Soc. Am. A.* 37 643–652. 10.1364/JOSAA.385832 32400549

[B12] Grossman N. Nikolic K. Toumazou C. Degenaar P. (2011). Modeling study of the light stimulation of a neuron cell with channelrhodopsin-2 mutants. *IEEE Trans. Biomed. Eng.* 58 1742–1751. 10.1109/tbme.2011.2114883 21324771

[B13] Huidobro N. De la Torre-Valdovinos B. Mendez A. Treviño M. Arias-Carrion O. Chavez F. (2018). Optogenetic noise-photostimulation on the brain increases somatosensory spike firing responses. *Neurosci. Lett.* 664 51–57. 10.1016/j.neulet.2017.11.004 29128628

[B14] Huidobro N. Mendez-Fernandez A. Mendez-Balbuena I. Gutierrez R. Kristeva R. Manjarrez E. (2017). Brownian optogenetic-noise-photostimulation on the brain amplifies somatosensory-evoked field potentials. *Front. Neurosci.* 11:464 10.3389/fnins.2017.00464 PMC5583167 28912671

[B15] Jackman S. L. Chen C. H. Regehr W. G. (2019). *In vivo* targeted expression of optogenetic proteins using silk/AAV films. *J. Vis. Exp.* 144:e58728. 10.3791/58728 30882792

[B16] Lebedev M. A. Opris I. Casanova M. F. (2018). Editorial: augmentation of brain function: facts, fiction and controversy. *Front. Syst. Neurosci.* 12:45 10.3389/fnsys.2018.00045 PMC6143785 30258355

[B17] McDonnell M. D. Iannella N. To M. S. Tuckwell H. C. Jost J. Gutkin B. S. (2015). A review of methods for identifying stochastic resonance in simulations of single neuron models. *Network* 26 35–71. 10.3109/0954898x.2014.990064 25760433

[B18] McDonnell M. D. Ward L. M. (2011). The benefits of noise in neural systems: bridging theory and experiment. *Nat. Rev. Neurosci.* 12 415–426. 21685932 10.1038/nrn3061

[B19] Medina L. E. Lebedev M. A. O’Doherty J. E. Nicolelis M. A. (2012). Stochastic facilitation of artificial tactile sensation in primates. *J. Neurosci.* 32 14271–14275. 10.1523/jneurosci.3115-12.2012 23055496 PMC3502008

[B20] Moss F. Ward L. M. Sannita W. G. (2004). Stochastic resonance and sensory information processing: a tutorial and review of application. *Clin. Neurophysiol.* 115 267–281. 10.1016/j.clinph.2003.09.014 14744566

[B21] Nikolic K. Jarvis S. Grossman N. Schultz S. (2013). Computational models of optogenetic tools for controlling neural circuits with light. *Conf. Proc. IEEE Eng. Med. Biol. Soc.* 2013 5934–5937. 24111090 10.1109/EMBC.2013.6610903

[B22] Pawela C. DeYoe E. Pashaie R. (2016). Intracranial injection of an optogenetics viral vector followed by optical cannula implantation for neural stimulation in rat brain cortex. *Methods Mol. Biol.* 1408 227–241. 10.1007/978-1-4939-3512-3_15 26965126

[B23] Remedios L. Mabil P. Flores-Hernández J. Torres-Ramírez O. Huidobro N. Castro G. (2019). Effects of short-term random noise electrical stimulation on dissociated pyramidal neurons from the cerebral cortex. *Neuroscience* 404 371–386. 10.1016/j.neuroscience.2019.01.035 30703508

[B24] Shin Y. Yoo M. Kim H.-S. Nam S.-K. Kim H.-I. Lee S.-K. (2016). Characterization of fiber-optic light delivery and light-induced temperature changes in a rodent brain for precise optogenetic neuromodulation. *Biomed. Opt. Express* 7 4450–4471. 10.1364/BOE.7.004450 27895987 PMC5119587

[B25] Van der Groen O. Wenderoth N. (2016). Transcranial random noise stimulation of visual cortex: stochastic resonance enhances central mechanisms of perception. *J. Neurosci.* 36 5289–5298. 10.1523/JNEUROSCI.4519-15.2016 27170126 PMC6601807

[B26] Zelena D. Demeter K. Haller J. Balázsfi D. (2017). Considerations for the use of virally delivered genetic tools for in-vivo circuit analysis and behavior in mutant mice: a practical guide to optogenetics. *Behav. Pharmacol.* 28 598–609. 10.1097/fbp.0000000000000361 29099403

